# Main Outcomes of the HEBE Trial: Improving Cardiorespiratory Fitness and Body Composition Through a Tailored Feasible Lifestyle Program

**DOI:** 10.3390/nu18121918

**Published:** 2026-06-12

**Authors:** Daniela Lucini, Federica Rota, Giuseppe Marano, Gianluigi Oggionni, Ester Luconi, Simona Iodice, Francesca Bianchi, Chiara Mandò, Giuseppina Bernardelli, Mara Malacarne, Silvana Castaldi, Patrizia Boracchi, Valentina Bollati, Mario Clerici, Elia Mario Biganzoli, on behalf of the HEBE Consortium

**Affiliations:** 1 Department of Medical Biotechnology and Translational Medicine, Department of Excellence 2023–2027, University of Milan, 20129 Milan, Italy; gianluigi.oggionni@unimi.it (G.O.); mara.malacarne@unimi.it (M.M.); 2 Exercise Medicine Unit, IRCCS Istituto Auxologico Italiano, 20135 Milan, Italy; g.bernardelli@unimi.it; 3 Department of Clinical Sciences and Community Health, Department of Excellence 2023–2027, University of Milan, 20122 Milan, Italy; federica.rota@unimi.it (F.R.); simona.iodice@unimi.it (S.I.); 4 Department of Biomedical and Clinical Sciences, University of Milan, 20122 Milan, Italy; giuseppe.marano@unimi.it (G.M.); ester.luconi@unimi.it (E.L.); chiara.mando@unimi.it (C.M.); patrizia.boracchi@unimi.it (P.B.); valentina.bollati@unimi.it (V.B.); elia.biganzoli@unimi.it (E.M.B.); 5 Department of Biomedical Science for Health, University of Milan, 20133 Milan, Italy; francesca.bianchi1@unimi.it (F.B.); silvana.castaldi@unimi.it (S.C.); 6 Laboratorio Morfologia Umana Applicata, IRCCS Policlinico San Donato, 20097 Milan, Italy; 7 Fondazione IRCCS Ca’ Granda Ospedale Maggiore Policlinico, 20122 Milan, Italy; 8 INES (INitiative of Epigenetics for Smiles), Università Degli Studi di Milano, 20122 Milan, Italy; 9 Department of Pathophysiology and Transplantation, University of Milan, 20122 Milan, Italy; mario.clerici@unimi.it; 10 Don C. Gnocchi Foundation, Istituto di Ricovero e Cura a Carattere Scientifico (IRCCS) Foundation, 20148 Milan, Italy

**Keywords:** nutrition, body composition, cardiorespiratory fitness (CRF), VO_2max_, metabolic equivalents (METs), healthy ageing, lifestyle, physical exercise, inflamm-ageing, prevention

## Abstract

**Background/Objectives:** Lifestyle Modification Programs (LMPs) based on exercise and nutrition aim to prevent/manage chronic diseases and foster well-being. However, moving LMPs from research to medical practice can be challenging, as programs must be both effective and feasible. The primary goal of this study was to assess cardiorespiratory fitness (CRF) changes according to an LMP, measured through VO_2max_, as a key indicator of health outcomes and intervention efficacy. **Methods:** In this single-arm intervention study, 100 subjects were enrolled; per-protocol analysis of main parameters was performed on 85 participants (15 were excluded due to medical/technical reasons). A feasible intervention program (of low resource intensity with only two physician/patient encounters) provided personalized exercise prescription, optimized nutritional habits based on the Mediterranean diet and Healthy Eating Plate principles, and supported behaviour change. We assessed CRF through VO_2max_, a key indicator of health outcomes and intervention efficacy. We also analyzed, using regression analysis, the relationship between VO_2max_ (the gold-standard measure of CRF) and MET_Speak_, a simpler, feasible parameter of CRF derived from Exercise Stress Testing. Body composition (BC) and AHA diet score were also measured at baseline and post-6-month intervention. Statistical analyses included paired comparisons and multivariable regression to explore factors influencing CRF changes. **Results**: Analysis on the primary outcome, VO_2max_, was performed according to the intention-to-treat principle and per-protocol. This feasible protocol resulted in a significant increase in VO_2max_, improvements in fat-free mass, and a reduction in fat mass. Overall, 42.4% of participants achieved an improvement of ≥1 MET, a change previously associated with reduced mortality risk. Older participants tend to experience smaller improvements in VO_2max_. **Conclusions**: Although observing an improvement in CRF and BC following an LMP is not surprising, the strength of the study is to show the feasibility of implementing an effective, feasible LMP into clinical routine, supporting the integration of such programs into clinical practice.

## 1. Introduction

Physical activity/exercise, along with healthy nutrition, is widely recognized as a fundamental tool to foster well-being, prevent and manage chronic non-communicable diseases (CNCDs), and improve quality of life at all ages [1,2,3,4,5]. It is endorsed in international guidelines for its ability to prevent more than 70% of cardiometabolic diseases and over 40% of cancer-related deaths. It directly acts on multiple control mechanisms, reducing systemic inflammation, promoting immune, metabolic and autonomic regulation [5,6,7,8,9], improving cardiorespiratory fitness (CRF) [10,11,12,13,14], and modulating “inflamm-ageing” [15,16].

Lifestyle Modification Programs (LMPs) based on exercise and healthy nutrition represent, therefore, a promising approach to prevent/manage CNCDs [17,18,19,20]. However, to move from research settings to routine clinical practice, these programs must be both efficacious, capable of improving underlying pathogenetic mechanisms, and feasible, ensuring accessibility and cost-effectiveness. Although multifactorial and tailored, supervised programs are often the most effective [18,19,20], they are typically resource-intensive and time-consuming, requiring financial investment and multidisciplinary interventions. Conversely, overly simplistic programs, based solely on general lifestyle counselling, frequently fail to produce meaningful results due to a lack of clear clinical goals and reliable outcome markers [21,22].

CRF represents a gold-standard marker for monitoring the effectiveness of LMPs [23,24]. It directly reflects aerobic capacity (Volume of Maximal Oxygen uptake (VO_2max_) assessed through a cardiopulmonary exercise test (CPX)), and it is often expressed in metabolic equivalents (METs), which represent multiples of the basal rate of oxygen consumption at rest, a derived practical, standardized measure of physical fitness [14,25,26] frequently used in clinical practice. Both VO_2max_ and METs at exercise peak (MET_Speak_) are inversely and strongly associated with all-cause mortality [11,12,13,14,16,25,26]. Recent evidence has demonstrated that even a modest improvement in CRF, such as an increase of 1 MET from the initial assessment, is associated with a significant reduction in mortality risk, regardless of baseline CRF levels [11].

This study was conducted as part of the HEBE Project (Healthy Aging Versus Inflamm-Aging: The Role of Physical Exercise in Modulating the Biomarkers of Age-Associated and Environmentally Determined Chronic Diseases), an ongoing initiative funded by the University of Milan. HEBE is a pre–post single-arm clinical trial, initiated in 2022, involving over 100 researchers working collaboratively to explore the role of physical exercise in modulating inflamm-ageing and its impact on health and CNCD risk [27]. The project includes 10 Lines of Research; this study is part of Line 1 (“proof of concept”), which aimed to evaluate the feasibility of implementing, into clinical routine, an effective, well-accepted LMP, requiring low economic resources, thereby overcoming the main organizational and economic barriers usually encountered when transitioning LMPs from research settings to clinical practice.

The primary goal of this study was to assess CRF changes according to the LMP, measured through VO_2max_, as a key indicator of health outcomes and intervention efficacy, implementing a feasible clinical protocol based on exercise prescription, complemented by optimization of nutritional habits, stress management, smoking cessation, sleep hygiene, and support for behaviour change, with the potential for integration into routine clinical practice.

## 2. Materials and Methods

### 2.1. Study Design

**Figure 1:**
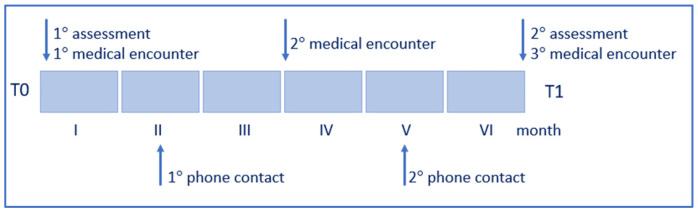
Timeline of assessments, medical encounters, and follow-up activities over the six-month intervention.

This is a six-month interventional trial involving a single group of 100 participants (registered in Clinicaltrials.gov (NCT05815732) 14 April 2023—retrospectively registered, https://clinicaltrials.gov/study/NCT05815732 accessed on 1 June 2026) that has been previously described, including the details of the power calculation [27]. We previously published the entire protocol [27] and the methodology for participant selection. Figure 1 reports the timeline of assessments, medical encounters, and follow-up activities of the six-month interventional trial. Sample size was based on the primary endpoint, namely the change in VO_2max_ during the observation period. Assuming a two-sided paired Student’s *t*-test with a significance level of α = 0.05, a sample of 100 paired pre- and post-intervention observations provided 80% power to detect an effect size of d = 0.28, defined as the mean difference divided by the standard deviation of the paired differences. According to Cohen’s criteria, this effect size lies between small (0.2) and medium (0.5) magnitude effects and was considered appropriate for the objectives of the study.

All University of Milan employees were contacted via email and invited to complete a lifestyle questionnaire, which also included a request to indicate their willingness to participate in the personalized intervention program. Briefly, a total of 983 participants, representing 21.9% of the University of Milan workforce, completed the initial lifestyle assessment questionnaire. Three hundred and ninety individuals indicated interest in taking part in the program. From this pool, 100 subjects who met the inclusion criteria [27] (age 18–70 years, no competitive physical activities, absence of any main cardiometabolic, oncologic, neurodegenerative or psychiatric conditions within the past three years) were stratified into groups based on the following criteria: (i) age—50% younger than 50 years and 50% aged 50 years or older; (ii) sex—50% female and 50% male; and (iii) Body Mass Index (BMI)—50% with BMI < 25 and 50% with BMI ≥ 25. Resulting from the above criteria, a total of 8 strata were formed. The strata were balanced with respect to the predefined criteria, and, within each stratum, subjects were enrolled sequentially according to their response date. In the event of exhaustion of the available participants within a specific stratum, additional participants were recruited following the order of their expression of interest.

Each subject followed a personalized physical exercise program and optimization of nutritional habits, with clinical and biological assessments performed at baseline (T0) and at the end of the intervention (T1). Informed consent was obtained from all individuals participating in the study. The study protocol adhered to the ethical principles outlined in the Declaration of Helsinki and complied with Title 45 of the U.S. Code of Federal Regulations, Part 46, Protection of Human Subjects, revised on 13 November 2001, and effective as of 13 December 2001. The HEBE study received ethics approval from the University of Milan’s Ethical Committee (Approval No. 62/22, dated 30 June 2022) and the IRCCS Istituto Auxologico’s Ethical Committee (Approval Code: 2022071912, dated 27 July 2022). At baseline (T0), all participants signed an agreement consenting to the use of their anonymized data for population studies and potential publications. Participants confirmed that they understood they could not be identified through any published results, as all data were fully anonymized by the authors.

### 2.2. Assessments

All subjects underwent the following assessment, both at T0 (baseline) and T1 (after 6 months of intervention):

- Medical history and physical examination, including anthropometric measurements (body weight, height, and waist circumference) and hemodynamics (resting systolic and diastolic blood pressure, heart rate). Biological sampling included fasting plasma glucose and lipid profile. Based on clinical and anthropometric measurements, metabolic syndrome was defined according to guidelines [28].
- Body composition was assessed using bioelectrical impedance analysis [29] (BIA, Bodystat Quadscan 4000, BodyStat Ltd., Sulby, Isle of Man, British Isles, UK), providing estimates of fat mass (FM) and fat-free mass (FFM). The Fat Mass Index (FMi) and Fat-Free Mass Index (FFMi) were calculated to normalize FM and FFM values for height, using the following formulas: FMi = FM (kg)/height (m^2^); FFMi = FFM (kg)/height (m^2^).
- Lifestyle

An ad hoc questionnaire was employed to quantify lifestyle [30,31,32,33,34,35]. Physical activity (total activity volume) was assessed using the International Physical Activity Questionnaire (IPAQ) [36], as were sedentariness, sleep, smoking, alcohol consumption, stress, and somatic symptoms perception. The quality of nutrition was assessed using the American Heart Association Healthy Diet Score (AHA score) [37], which considers the consumption of fruits/vegetables, fish, sweetened beverages, whole grains, and sodium. Appendix A.1 reports the details of the lifestyle assessment.

- Cardiopulmonary Fitness Testing

Aerobic fitness, the primary outcome of the study, was assessed through maximal oxygen consumption (VO_2max_) [24]. A standard incremental cycle ergometer test (Custo Cardio 300, Ottobrunn, Germany; VYNTUS CPX, Hoechberg, Germany) was performed, with a duration of approximately 10 ± 2 min, to ensure that a respiratory exchange ratio (RER) of ≥1.1 was achieved. During the test, heart rhythm and frequency were monitored using a 12-lead ECG, and ventilatory and gas-exchange parameters were analyzed breath-by-breath. The VO_2max_ was calculated as the highest oxygen uptake achieved during the test. Additionally, the peak METs reached (MET_Speak_) were determined using the following formula [38]:

Peak MET = 1 + (12 × Work)/(3.5 × Weight),

where Work is the maximal workload (watts) and Weight is body weight (kg).

We also performed other functional tests to complement VO_2max_: the 6-min walking test (6MWT) was performed following international guidelines [39], and handgrip strength (HGS) was used to assess muscular strength using isometric dynamometry [40], in a sitting position, with the elbow bent at a 90-degree angle, repeated three times for both the right and left arms. We considered the average of the three readings.

### 2.3. Lifestyle Prescription Program

At T0, all enrolled subjects received a tailored exercise prescription and optimization of nutritional habits, made by a physician who has specific certified training in internal medicine and clinical psychology, following recent guidelines [1,3,41]. Table 1 reports the main steps of the first visit (T0). The specific methodology followed for prescription has already been described [20]. Briefly, in the first encounter, after having established an empathic relationship with the subject, considering their expectations of the program, the analysis of the lifestyle assessment and of the clinical test results permitted the subject’s clinical status to be shown and specific clinical goals to be defined. Then, cognitive resources were given to translate into practical action the possibility of realizing the desired behavioural change (for instance, how to select the best moment in the day to perform exercise, or how to select a realistic modality of exercise). Subsequently, the specific exercise prescription was presented. In particular, it was discussed how to follow and check the training heart rate, how to overcome common barriers, and how to perceive the initial benefit granted by the intervention. In the second visit, after a simple medical assessment of weight, waist circumference, blood pressure and basal heart rate, the obtained results and barriers encountered were discussed using a problem-solving approach. The subject was encouraged to ask for more details on how to improve behaviour and to discover the required cognitive and practical resources that best account for their needs, raising their awareness of the consequences of the new behaviour on their quality of life and well-being. Moreover, the subject was helped in defining further steps towards the long-term goal, regaining control of their life and well-being.

#### 2.3.1. Exercise Prescription

The exercise prescription provided a clear definition of the modality, intensity, frequency, duration, and progression of exercise, and was based on individual characteristics, limitations, and preferences. It aimed to improve cardiorespiratory fitness, and the volume of prescribed endurance aerobic exercise was at least the volume recommended by international guidelines [1,3] to improve health, corresponding to at least 150 min of aerobic endurance activity per week. This volume could be exceeded as long as it was within 300 min of aerobic endurance activity per week. Training heart rate was calculated using the Heart Rate Reserve Formula [(Maximal Heart Rate − Resting Heart Rate) × %value of exercise intensity) + Resting Heart Rate] following more recent guidelines [3], verifying that it was below the anaerobic threshold, estimated by a CPX. Moreover, flexibility and strength exercises were prescribed according to individual characteristics to improve joint flexibility and muscular strength (accompanied by video tutorials), and counselling to reduce sedentary time was provided. Subjects independently decided, after a specific discussion with the physician, where to perform the prescribed exercise (indoors—at a gym or at home—or outdoors, or both), and training sessions were unsupervised. A wearable device (Fitbit Inspire 3) was employed to check training HR during exercise.

#### 2.3.2. Nutrition

No specific prescription for a nutritional program was made, as the main focus of the study was on the exercise prescription. We only considered an optimization of nutritional habits, which was guided by the principles of the Mediterranean diet and the Healthy Eating Plate. In particular, participants were encouraged to structure their meals according to balanced proportions: approximately half of the plate composed of vegetables and fruits, one quarter of whole grain carbohydrates, and one quarter of protein sources (such as unprocessed white meat, fish, dairy, eggs, legumes, and nuts), highlighting the importance of portion balance among food groups. All subjects were advised to consume 0.8–1.0 g of protein per kilogram of body weight, to prefer extra virgin olive oil as the main source of added fat, and to prioritize whole grains, fruits, and vegetables in their daily diet [42]. While participants were free to choose specific foods, they were instructed to adhere to these general principles of dietary composition and balance.

#### 2.3.3. Other Lifestyles

We also provided written guidance on proper nutrition and other healthy lifestyles through materials available on the HEBE website (https://hebe.unimi.it, accessed on 1 June 2026) [1,2,3,6,17,28,37,42]. This platform was specifically developed and curated by researchers from the University of Milan with expertise in the relevant fields, ensuring that all recommendations and information are grounded in scientific evidence and evidence-based medicine. A cognitive–behavioural approach was employed to enhance participants’ motivation [17,43,44]. The intervention was further supported by guidance on stress management and smoking cessation.

Additionally, participants attended a third medical encounter dedicated to reviewing results, receiving feedback, and discussing future recommendations.

At T1, participants’ overall satisfaction with the program and the perceived difficulty in adhering to it were assessed using rating scales from 0 (“lowest satisfaction” or “lowest difficulty”) to 10 (“highest satisfaction” or “highest difficulty”) for each measure. Additionally, participants evaluated their perceived well-being by comparing their condition before and after the intervention, choosing among the following options: “much worse,” “worse,” “no change,” “improved,” or “much improved [45].

Appendix A.2 provides details on additional parameters collected, the analysis of which will be reported in future publications.

### 2.4. Outcomes

The primary outcome was the change in VO_2max_ (aerobic capacity) from baseline to six months, while the secondary outcomes were changes in body composition and other functional parameters (including variables derived from CPX/exercise stress test, 6 min walking test, handgrip and blood examination).

### 2.5. Statistical Analysis

Categorical variables were summarized using counts and percentages. For each numerical variable, distributions at baseline, post-intervention, and pre–post changes were examined using histograms, and outliers were identified. Because several variables showed skewed distributions and/or outlying values, numerical variables were generally summarized using the median and interquartile range (25th–75th percentiles).

#### 2.5.1. Analysis of the Main Outcome

The primary outcome (pre–post change in VO_2max_) showed an approximately symmetric distribution and no relevant outliers. Inspection of the histogram and normal Q–Q plot further indicated that the distribution was reasonably consistent with normality. Therefore, the effect of the lifestyle program was assessed using a paired *t*-test (two-tailed, α = 0.05), and the respective 95% confidence interval for the mean pre–post difference was also calculated.

In addition, factors associated with a greater or lesser change in VO_2max_ were investigated using multivariable linear regression models (ordinary least squares). The dependent variable was the pre–post change in VO_2max_. Covariates were selected a priori and included: baseline VO_2max_, gender, age, volume of vigorous and moderate physical activity (<600 vs. ≥600 MET × min/week), AHA score (0–1, 2–3, 4–5), perceived stress score (0–10 Likert scale), metabolic syndrome (yes/no), fat-free mass (%) and overall satisfaction with the program (0–10 Likert scale). Continuous variables were modelled as linear terms, whereas categorical variables were represented through indicator variables. Model assumptions were assessed using graphical diagnostics, including evaluation of the linearity of continuous covariate effects, homoscedasticity, and approximate normality of residuals.

The analyses described above were based on the intention-to-treat principle. Therefore, all available participant data were used, and missing values were handled using multiple imputation [46,47,48] by chained equations (MICE) [49,50] under the missing-at-random (MAR) assumption. In this approach, missing values are iteratively estimated using regression models based on the information available from complete variables.

The imputation model included the primary outcome, all covariates described above, and the sampling strata. Linear regression models were used to impute continuous variables, whereas logistic regression models were used for binary and categorical variables.

Twenty imputed datasets were generated, exceeding the percentage of missing data in the primary outcome, as recommended in the literature [50]. Estimates obtained from the imputed datasets were combined using Rubin’s rule [48]. In addition, the same analysis was performed only on participants who completed the treatment (per-protocol analysis).

#### 2.5.2. Analysis of the Secondary Outcomes

All analyses of secondary outcomes were exploratory and were performed on participants who completed the program (per-protocol sample). This approach was adopted to evaluate the effect of the intervention among participants who adequately adhered to the exercise program. Consistent with TREND recommendations [51], results were primarily interpreted through effect estimates and their precision rather than through hypothesis testing.

Confidence intervals at the 95% level of the median pre–post difference and the relative difference between proportions were reported, respectively, for numerical and categorical outcomes. For numerical outcomes, the CIs were obtained using quantile regression [52], with standard errors obtained via bootstrap resampling (1000 replications).

For categorical outcomes, the relative difference was calculated as the percentage change between post-treatment and pre-treatment proportions, i.e., 100 × (p_1_ − p_0_)/p_0_, where p_1_ and p_0_ denote the post-treatment and pre-treatment proportions, respectively.

Confidence intervals were adjusted for multiple comparisons (49 outcomes) using the Bonferroni correction, resulting in an overall confidence level of approximately 99.9%.

Differences were considered statistically significant when the corresponding confidence interval did not include zero.

The potential bias attributable to the exclusion of participants who discontinued the program was investigated by comparing the distributions of baseline characteristics between completers and non-completers. The comparisons were performed on a restricted set of representative demographics (age, gender), lifestyle indicators (physical activity, AHA score, perceived stress score, metabolic syndrome) and test parameters (percentage of fat-free mass, VO_2max_). For categorical variables, Cramer’s V index was calculated; this index ranges from 0 to 1, with values close to 0 indicating similarity between the two groups. For numerical variables, the distributions in the completers group were represented using boxplots, and the corresponding values for non-completers were overlaid. Good overlap between these distributions indicates comparability between the two groups.

The correlation between VO_2max_ and peak METs was assessed using scatterplots and Pearson’s correlation coefficient (r). Linear regression was used to quantify the expected change in VO_2max_ associated with a 1-MET increase in peak METs. Finally, the impact of the stratified sampling design on the results was assessed through a sensitivity analysis using sampling weights [50]. All analyses were conducted in R version 4.4.0 [53] using the packages vtable [54], mice [55], and survey [56].

## 3. Results

### 3.1. Population

**Figure 2:**
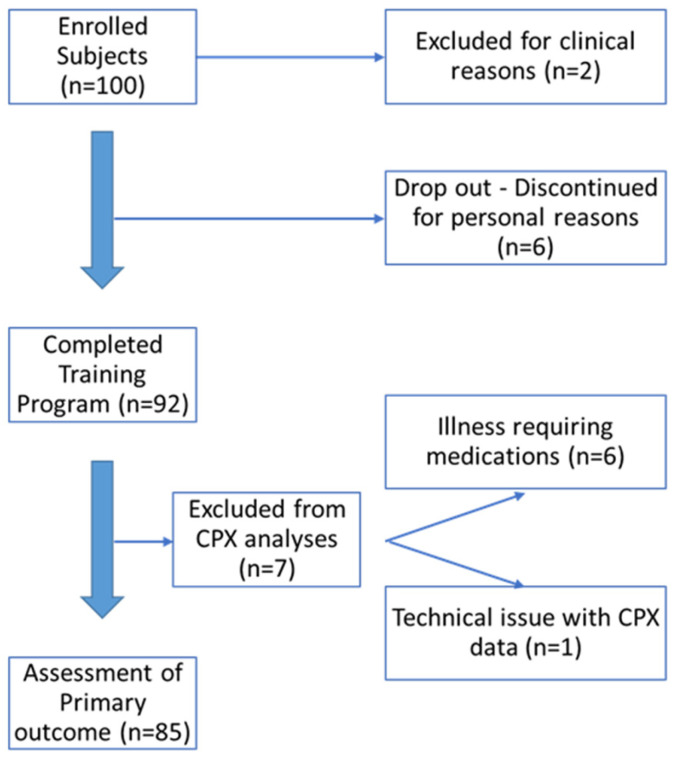
Flowchart of study participants from T0 to T1, including outcome analysis.

Among the 100 participants enrolled, two were excluded following a post-enrollment assessment that revealed ineligibility (Figure 2). The reasons for exclusion included medical conditions identified during the initial evaluation that were deemed incompatible with the intervention. The baseline characteristics of the remaining 98 are reported in Appendix A, Table A1 and Table A2. Among the 98 participants who underwent the physical exercise program (LMP), six voluntarily discontinued the program for personal reasons (Figure 2). These 98 were included in the intention-to-treat analysis, and 92 in the per-protocol analysis, except for the cardiopulmonary exercise test (CPX) parameter analysis. In fact, in the latter case, six further participants were excluded because they developed illnesses during the intervention that required medical treatment, as prescribed by their general practitioners, using medications that could significantly influence the measured parameters (e.g., beta-blockers). Furthermore, one participant was excluded due to technical issues encountered during the analysis of cardiopulmonary exercise (CPX) test data. In conclusion, per-protocol analysis of CPX parameters was performed on 85 participants. We report blood test results (fasting plasma glucose and lipid profile), perception of satisfaction and difficulty of the program and lifestyles parameters in Appendix A (respectively Table A2, Table A3, Table A4 and Table A5). We reported the summary of primary outcome in Table 2, while the secondary outcomes are summarized in Table 3 and Table 4.

At baseline, the maximum proportion of missing data was 4/92 for lifestyle indicators (4.3%, Table A4 and Table A5), 2/92 for cardiopulmonary parameters, reported in Table 3, 1/92 for body measurements, reported in Table 4, and no missing values for blood test parameters (Table A3). After the completion of the program, the highest proportion of missing data was obtained in 9/92 participants who did not report alcohol consumption (Table A5).

The overall satisfaction with the program was high, with at least 75% of the participants’ scores being 8/10 or more (1st quartile), and 83.7% of the participants declared that the overall perception of well-being was improved or much improved (see Table A3).

### 3.2. Main Outcome: Cardiopulmonary Test

The mean pre–post difference in VO_2max_ estimated by intention-to-treat analysis was 2.86 mL/kg/min, with a 95% CI of 2.23 to 3.55 mL/kg/min, showing statistical evidence of improvement in aerobic fitness (*p* < 0.0001, Table 2). Similar results were obtained in the per-protocol sample, with a mean pre–post difference equal to 2.95 mL/kg/min and a 95% CI of 2.32 to 3.57 mL/kg/min (*p* < 0.0001). These results are consistent with those obtained by sensitivity analysis (Table 2).

### 3.3. Secondary Outcomes

#### 3.3.1. Functional Parameters

At T1, significant improvements were observed in several key cardiopulmonary and functional parameters compared to T0 (Table 3). Statistically significant changes are indicated, showing improvements in VO_2max_, MET_Speak_, anaerobic threshold (AT) parameters, workload, and 6MWT performance.

Considering the overall group, MET_Speak_ improved from 8.5 to 9.4 (Δ 0.7, 95% CI: 0.3, 1.1). MET_Speak_ increased with a median difference of 0.7 (95% CI: 0.3 to 1.1). Notably, 42.4% of participants achieved an improvement of ≥1 MET, a change previously shown to be associated with a lower mortality risk [11], even though we may not argue a specific reduction in mortality risk in our population.

Improvements were also observed in AT % peak VO_2_, AT VO_2_ (mL/min/kg), AT Load (Watt), and peak Load (Watt). Functional capacity, assessed via the 6MWT, improved significantly, with distances increasing with a median difference of 27.0 m (95% CI: 13.5 to 47.5).

**Figure 3:**
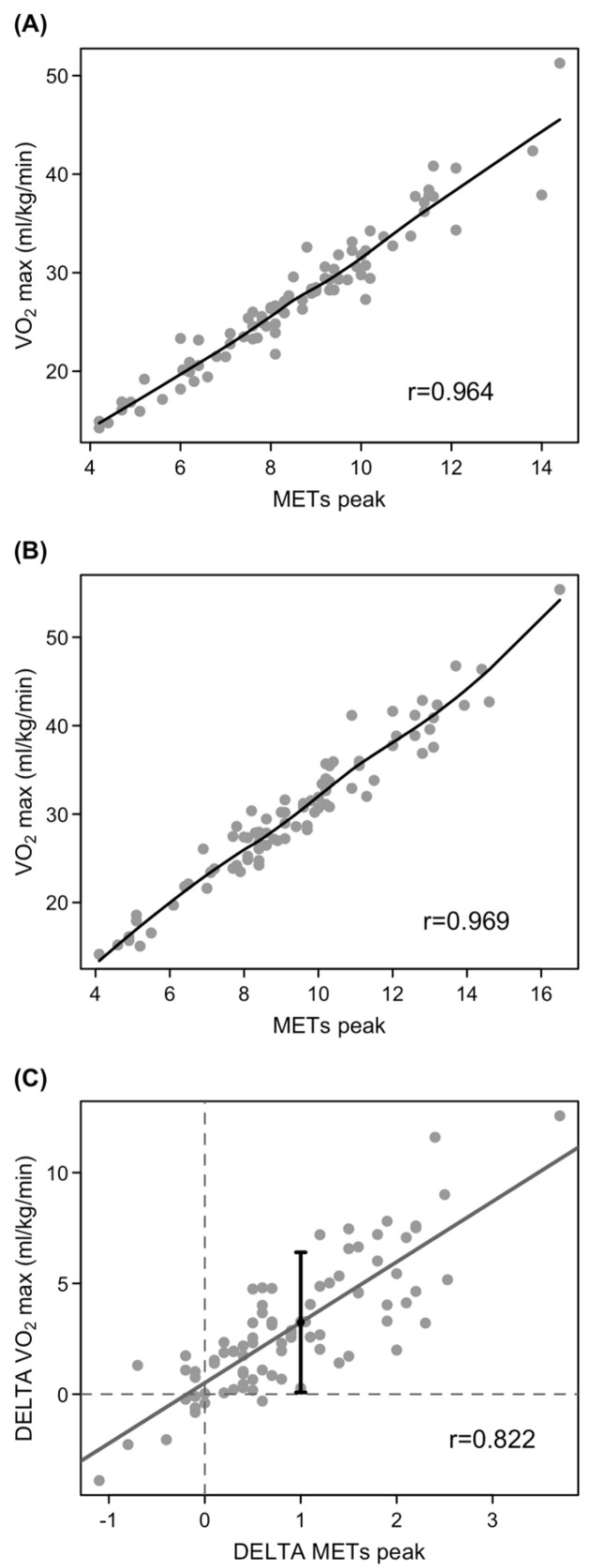
Correlation and regression analysis between VO_2max_ and MET_Speak_. Panel (**A**): scatterplot between measurements at baseline (T0) of VO_2max_ and MET_Speak_; panel (**B**): scatterplot between measurements at program completion (T1) of VO_2max_ and MET_Speak_; panel (**C**): scatterplot and regression line between pre–post differences (DELTA) of the same variables. The black curves in panels (**A**,**B**) were obtained using a nonparametric regression (lowess smoother). The black line in panel (**C**) is the regression line, and the interval is the prediction interval for delta VO_2max_ conditional on DELTA MET_Speak_ = 1.

Figure 3 shows a strong positive correlation between VO_2max_ and MET_Speak_ at baseline (Figure 3A) and at the end of the program (Figure 3B), with correlation coefficients of r = 0.964 and r = 0.969, respectively. This indicates a very strong association between maximal oxygen uptake and metabolic equivalents at peak exercise. Furthermore, the observed improvements in VO_2max_ and MET_Speak_ after the intervention support the effectiveness of the lifestyle management program in enhancing cardiorespiratory fitness. Figure 3C shows the pre–post differences in these parameters. The estimate of the average pre–post difference in VO_2max_ conditional on a 1-unit increase in MET_Speak_ is 3.24 mL/min/kg, with a 95% prediction interval of 0.078 to 6.40 mL/min/kg. These results emphasize the proportional relationship between the improvements in VO_2max_ and MET_Speak_, strengthening the role of MET_Speak_ as a proxy of VO_2max_, both at baseline and in response to the intervention.

#### 3.3.2. Body Composition Changes

Body composition changes from T0 to T1 (Table 4) showed significant improvements in median fat mass (kg), median fat mass (%), fat-free mass (%), and total Body Water (%). We did not observe a reduction in fat-free mass (Kg). BMI showed a trend toward reduction, with a difference of −0.5 kg/m^2^ (95% CI: −0.95 to −0.05), although the corresponding CIobtained within sensitivity analysis did not confirm the statistical significance of this result. Changes in waist circumference were statistically significant, with a difference of −3.0 cm (95% CI: −5.8 to −0.2); however, as above for BMI, the sensitivity analysis did not confirm statistical significance.

#### 3.3.3. Multivariable Regression Model

**Figure 4:**
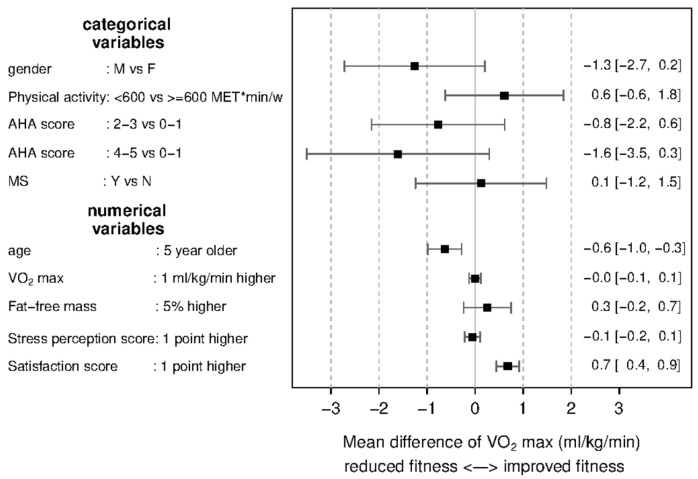
Forest plot showing effect estimates and respective 95% CIs from multivariable regression model. Black squares represent the estimates of regression coefficients from the multivariate regression model and the straight lines represent the respective 95% CIs The regression coefficients provide estimates of mean between-group differences in delta VO_2max_ (for categorical variables) or the mean change in delta VO_2max_ associated with a pre-fixed increase in the respective variable (numerical variables). For example, the mean ΔVO_2max_ is −1.3 mL/kg/min lower in males versus females (categorical variable), with a 95% CI of −2.7 to 0.2 mL/kg/min. In a similar fashion, the mean ΔVO_2max_ of two subjects with a 5-year difference in age (numerical variable) is −0.6 mL/kg/min lower in the elder one, with a 95% CI of −1.0 to −0.3 mL/kg/min. MS = metabolic syndrome.

To investigate the factors influencing the change in VO_2max_ (delta VO_2max_), a multivariable regression model was estimated, with the following explanatory variables: age, gender, baseline VO_2max_ (VO_2max_ at T0), and program satisfaction. The estimates of the effects of these variables are shown in Figure 4. In detail, age was negatively associated with delta VO_2max_, with an estimated mean decrease of −0.63 mL/kg/min per 5-year increase in age (95% CI: −0.99 to −0.28) (adjusted for all the other variables in the regression model, described above). This estimate indicates that older participants tend to experience smaller improvements in VO_2max_. Notably, program satisfaction was positively associated with delta VO_2max_, with an adjusted estimated mean 0.68-unit increase in delta VO_2max_ for every one-point increase in the perceived satisfaction score (95% CI: 0.44 to 0.92). The model estimates also suggest that worse lifestyle parameters (physical activity < 600 METs, or lower AHA diet scores) are associated with a greater estimated improvement in VO_2max_, even if these changes do not reach statistical significance.

#### 3.3.4. Other Lifestyle Components/Parameters

Table A4 and Table A5 reports differences in the other lifestyle components/parameters. Notably, the median difference in hours of sedentary behaviours was significantly reduced at T1. No significant differences were noted regarding changes before and after intervention regarding quality of nutrition (AHA score), alcohol consumption, sleep, perception of stress, fatigue and somatic symptoms, perception of quality of health, performance and sleep; nevertheless, a slight reduction in the perception of fatigue and stress and an improvement in the perception of quality of health, performance and sleep may be noted.

#### 3.3.5. Check of Non-Completers’ Data

**Figure A1:**
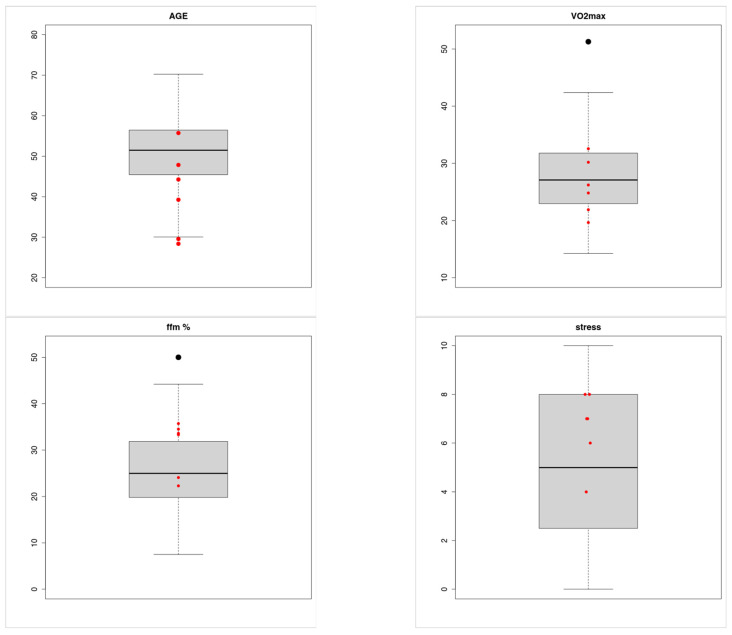
Distribution of numerical variables in completer and non-completer groups. The boxplots show the distributions of the pertinent variables in the completers, and the grey arrows in the non-completers.

Comparison between completers and non-completers showed similarity across most baseline characteristics. The main differences concerned gender and age distribution, with non-completers being more frequently male (5/6, 83.3%; versus 44.6% of completers; Table A6) and generally younger than participants who completed the program (Figure A1). Considering the limited number of subjects in the non-completer group, the relevance of these differences is not easy to assess.

## 4. Discussion

In this study, we observed that the applied lifestyle management program (LMP), based on tailored exercise prescription, optimization of nutritional habits, motivation to change and unsupervised training, was linked to improved body composition and increasing cardiorespiratory fitness in a cohort of university employees. Notably, 42.4% of participants achieved an improvement of ≥1 MET_Speak_, a change associated with a lower mortality risk [11], particularly among young participants.

### 4.1. Cardiorespiratory Fitness to Monitor LMPs

Clinical medicine encompasses a wide range of practices aimed at treating, managing, and preventing many CNCDs, as well as promoting overall well-being. Among these actions, lifestyle improvement may play an important role. However, translating LMPs from research settings into routine clinical practice remains challenging [57,58]. Two essential conditions must be met: interventions should effectively target mechanisms responsible for diseases to treat or prevent by defining clear clinical goals and reliable outcome markers, while also being feasible, accessible, and cost-effective. In this regard, the prescription of exercise, much like pharmacological therapy, requires individualized assessment and well-defined clinical goals to ensure its effectiveness [1,3,21,41,59]. Aerobic endurance exercise performed at moderate intensity for 150–300 min per week is required to improve CRF in adults, while strength training is necessary to improve muscular strength and mass [1,3]. The primary goal of this study was to assess CRF changes by an LMP, given that CRF is associated with reduced total mortality and improved cardiovascular and oncologic risk factors, psychosocial functioning, and well-being [10,11,12,13]. To achieve this goal, we prescribed exercise after cardiopulmonary exercise testing (CPX) to assess baseline CRF and determine training heart rates tailored to individual characteristics and clinical goals [1,3,20]. CPX is the gold standard for assessing CRF [24]; however, its use in clinical practice is often limited by cost and logistical challenges [11,14,23,26,60]. MET_Speak_ is, instead, simply derived from standardized exercise testing (a more commonly used clinical test), and it represents a practical measure of exercise capacity [11,26]. Notably, MET_Speak_ is inversely and strongly associated with all-cause mortality, with a 1-MET improvement linked to lower mortality risk [11]. Our analysis quantified the relationship between changes in VO_2max_ and MET_Speak_, showing that a 1-MET improvement corresponds to an average increase of 3.24 mL/min/kg in VO_2max_. This finding is not surprising, as the algorithms used to estimate both VO_2max_ and MET_Speak_ consider intertwined parameters; consequently, the strength of the correlation is not unexpected. Nevertheless, it may corroborate the usage of a simple parameter derived from a simple ECG stress test (MET_Speak_) as a practical proxy to estimate cardiorespiratory fitness in a clinical setting where more complex and costly evaluations, such as cardiopulmonary exercise testing, are not readily available.

A further point to consider is that another cardinal CPX variable, such as VE/VCO_2_ slope, which was within the normal range in our cohort, did not change after the intervention. This is physiologically consistent, as changes in VE/VCO_2_ slope are typically more evident in populations with cardiopulmonary disease, where ventilatory inefficiency is present and may improve with targeted interventions [61,62]. Hence, the improvements we observed in exercise capacity are more likely explained by peripheral and metabolic adaptations to training rather than by changes in ventilatory efficiency, which were already preserved at baseline [61,62].

In this study, the overall group of participants significantly improved CRF; notably, 42.4% of subjects achieved a clinically meaningful increase in 1 MET at peak exercise after the LMP, even though we may not argue a specific reduction in mortality risk in our population. We also observed that younger participants tended to experience greater improvements in VO_2max_. These data highlight that preventive strategies may be particularly effective in younger individuals, despite the fact that they may be less inclined to modify their behaviour, due to a perception of being healthy. While it is well established that young subjects exhibit a wide range of VO_2max_ values [63], less is known regarding the influence of age on improvements in cardiorespiratory fitness following an LMP. Ageing is associated with reduced cardiovascular reserve, impaired vascular function and diminished mitochondrial efficiency, all of which limit the magnitude of achievable improvements in VO_2max_ [64]. The available literature on this topic is limited and shows inconsistent findings [65,66,67]; therefore, our results may contribute to a better understanding of this field. It is important to note that these results were achieved without supervised training in a controlled environment, but rather through unsupervised training and self-managed meal preparation.

### 4.2. Body Composition

In addition, we observed a significant improvement in body composition, suggesting an important role of exercise in directly affecting metabolism and, at the same time, supporting the concept that physical activity and a well-balanced diet are most effective when combined within a comprehensive approach.

Interestingly, we did not observe a reduction in fat-free mass (Kg); rather, a slight, albeit non-significant, increase was detected. These data suggest that the LMP employed was effective in avoiding fat-free mass loss, which frequently characterizes these programs [68]. Nevertheless, we have to consider that bioelectrical impedance analysis has known limitations related to hydration status and measurement variability, which may call for caution in the interpretation of these results. Moreover, the lack of statistical significance in the reduction in waist circumference and BMI after sensitivity analysis suggests that the impact on central obesity should be considered with caution.

### 4.3. LMPs in Clinical Settings

The traditional approach to preventing several chronic diseases, based on the reduction in cardiometabolic risk through parameters such as lipid profile, blood pressure, and glucose level, is now increasingly complemented by strategies targeting the improvement of behaviours such as physical activity, healthy nutrition, stress management, smoking cessation, and good sleep hygiene (LMPs) [69,70]. This shift represents an important change in perspective, emphasizing feasible behaviour changes regardless of calculated cardiac risk [31], a concept particularly important for younger individuals, who are often characterized by low estimated cardiac risk. In this study, the observed results were achieved by employing an LMP which was highly feasible from both economic and organizational perspectives. The program involved only two in-person visits with an internal medicine physician, to prescribe exercise and provide guidance on proper nutrition (along with advice on stress management and smoking cessation). These were complemented by two phone consultations with a physiotherapist to support adherence and maintain motivation. A third, final medical encounter at the end of the program was dedicated to results review and motivation reinforcement. Participants were free to choose whether to perform exercise indoors (e.g., at home or in a gym) or outdoors, according to their preferences and logistical constraints. They were also free to decide the meal composition, while respecting the Mediterranean diet and Healthy Eating Plate principles. No specific recommendations were provided regarding the precise dosage of single macronutrients, with the exception of protein intake of 0.8–1.0 g per kilogram of body weight, derived from unprocessed white meat, fish, dairy products, eggs, legumes and nuts, as suggested by international guidelines [42].

A cognitive–behavioural approach was employed to motivate participants to adopt and maintain lifestyle changes (see Table 1) [17,43,44]. In particular, a patient empowerment approach [70] and the 5A model of implementing behavioural changes were considered, as recently recommended by medical scientific statements [71,72]. While specific cognitive–behavioural therapy may be more suitable for individuals with psychological impairments, training physicians in basic cognitive–behavioural techniques can improve the effectiveness and sustainability of LMPs [17,43,44,71,72]. In this study, we integrated this behavioural approach into routine internal medicine encounters with a patient, combining motivation techniques with traditional internal medicine practice. This strategy appears to be effective in empowering participants [68], even in the absence of chronic disease, and may be easily employed in a clinical setting [71,72]. Notably, sedentary habits were reduced, overall satisfaction with the program was positively associated with improvement in VO_2max_, and the employed statistical analysis suggests that worse lifestyle parameters (physical activity < 600 METs, or lower AHA diet scores) were associated with a greater estimated improvement in VO_2max_. Even if these latter changes did not reach statistical significance, they suggest that subjects characterized by unhealthy behaviour were motivated to improve their habits.

The importance of health literacy in influencing adherence to exercise programs needs to be discussed. It is well recognized [73] that an elevated level of health literacy facilitates the adoption of a healthy lifestyle, and that improving health literacy is a cornerstone of patients’ empowerment [70], particularly in non-supervised exercise programs, which need self-management capabilities [74]. Social research [75] found that a higher level of education in adults is linked to more physical activity.

In our study, we enrolled, by design, employees of the University of Milan (59.2% holding a PhD or postgraduate degree), and it may be argued that they have higher health literacy compared to the general population. Nevertheless, we have to consider that during the first medical visits, we dedicated time to improve health literacy, considering their level of knowledge and their clinical goals, as a pivotal strategy to increase their adherence to the program, independently of their educational degree. Obviously, socioeconomically diverse populations might require a different approach.

### 4.4. LMPs and Perceived Well-Being

An LMP represents one of the main strategies to improve overall well-being and reduce stress perception [76,77], as already observed in other studies [78,79], including studies conducted by our research group [31,32,33,34]. In this specific study, 83.7% of subjects who completed the program reported an improvement in their perceived personal well-being, and overall satisfaction with the program was high, with most participants rating the program at eight (on a 0–10 scale). Interestingly, 53.1% of participants reported a desire to increase their level of physical activity, while 22.4% expressed a willingness to adopt a healthier diet. The study did not unveil any significant differences in perceived stress, fatigue, or symptoms, although a slight improvement was observed. However, these findings should be interpreted with caution, given the limited sample size.

Similarly, the handgrip test showed no significant improvement in muscular strength, although an increase was observed in the reported frequency of strength and flexibility exercise sessions. These exercise modalities were also prescribed, along with video tutorials to support adherence. Nevertheless, we must consider that only strength exercises of moderate intensity were prescribed [1] to subjects without sarcopenia or muscular diseases, in combination with appropriate nutritional guidance, which may have contributed to the preservation of fat-free mass. The possibility of tailoring the progression of strength exercises with the help of an exercise physiologist might further improve muscle strength and mass, although this would likely increase the overall cost of the LMP.

### 4.5. Clinical Implications

The strength of the present study is to show the feasibility of implementing into clinical routine an effective, feasible and well-accepted LMP, thereby overcoming the main organizational and economic barriers usually encountered when transitioning LMPs from research settings to clinical practice. LMPs demonstrate substantial cost savings and elevated Returns On Investments [80,81]; on the other hand, the cost to perform them (due to the costs of many professionals, the cost of fitness centres, etc.) and organizational barriers may represent an issue to their realization in primary care settings. Our study required low economic resources (corresponding to the cost of three medical visits and the cost of a CPX or a less expensive stress ECG to tailor exercise prescription and to monitor results). Participants may decide where and how to perform exercise, based also on their own economic availability. This intervention model might offer significant scalability in different primary care settings and applicability to diverse populations.

### 4.6. Limitations

We have to recognize some limitations of our study, in particular, the following:

- It is a single-arm (pre–post) study, a condition which limits the isolation of the effect of the intervention from other experimental variables, unmeasured confounders, and potential observation effects such as the Hawthorne effect. Consequently, the findings should be interpreted with caution, and causal inferences cannot be established. Moreover, we do not have data on long-term follow-up data, and we may not demonstrate the maintenance of behavioural and physiological improvements in the long term.
- The sample size was calculated to detect a clinically relevant increase in VO_2max_ as the primary endpoint, and the follow-up was limited to six months due to funding constraints. In addition to the previous limitations, secondary outcomes were analyzed on a per-protocol basis, which may introduce selection bias and further reduce the effective sample size. Accordingly, inferences for secondary endpoints should be interpreted with caution. The stratified sampling plan ensured adequate representation of key subgroups and improved the precision of estimates within the target population. In addition, a weighted sensitivity analysis was performed to account for potential imbalances across strata, thereby enhancing the robustness of the findings. Nevertheless, the generalizability of the results to a broader population of healthy individuals remains limited, mainly by the study’s eligibility criteria and setting. In particular, the sample consists exclusively of employees of the University of Milan with a very high educational level (59.2% holding a PhD or postgraduate degree), limiting the generalizability of the results to populations with lower health literacy. Moreover, we did not assess the intervention effects after 6 months due to financial restraints.
- This paper focuses primarily on CRF and a subset of clinical variables commonly collected in trials. Other CPX variables were included in the text to provide a clinically meaningful, although not exhaustive, characterization of general cardiopulmonary status, without unnecessarily increasing methodological complexity or overextending the scope of the manuscript. We are planning another manuscript devoted to these data. Moreover, future analyses will explore the multi-omics biomarkers collected during the study to investigate interactions between immunological, metabolic, and autonomic control mechanisms.
- We mainly focused on exercise, which was tailored and prescribed, and it might be most responsible for the observed improvements; however, the counselling given on nutrition and other lifestyle components, albeit limited, might contribute to the observed results. Nevertheless, distinguishing the relative contribution of different lifestyle components may not have significant clinical relevance, considering that the preventive/therapeutic action of lifestyle improvement is more efficacious when all lifestyle components are considered and that improving one lifestyle component (in particular exercise) may also foster improvements in others [82].
- The retrospective registration of the trial may represent a limitation, considering that prospective registration is generally preferred for interventional studies
- We did not report in the present paper the data regarding the assessment of physical activity using a wearable device because we are preparing other papers on these specific (and other) data, considering that the HEBE study was designed to collect many markers and many different papers are planned. Anyway, this specific point was not a main goal of the present study, which focused on cardiorespiratory fitness, the strongest parameter affected by physical training, which may determine health outcomes.

## 5. Conclusions

This study provides evidence that implementing a feasible, cost-effective, and well-accepted LMP in routine clinical practice is feasible. The program induced significant improvements in CRF and body composition, highlighting its potential to support long-term health benefits. Tailored exercise prescription and optimization of nutritional habits, supported by limited physician encounters and digital resources, proved effective in achieving meaningful outcomes without imposing excessive costs or logistical challenges. The ability to integrate tailored LMPs into routine care with minimal time and economic investment represents a valuable opportunity for both patients and healthcare institutions, offering a scalable solution to promote health and prevent chronic diseases in clinical settings.

## Figures and Tables

**Table 1:** Overview of activities and assessments in the first medical visits.

1° VISIT ACTIONS	AIMS
Welcome	▪ Introduce the program and define the subject’s and physician’s roles & responsibilities ▪ Establish an empathic, maieutic relationship, understanding the subject’s concerns and circumstances
Clinical history and clinical assessment	▪ Definition of the subject’s health status ▪ Collection of anthropometric, hemodynamic data and general clinical assessment ▪ Analysis of lifestyle assessment ▪ Analysis of performed tests
Explanation of diagnosis and specific benefits derived from lifestyle change	▪ Make the subject aware of their clinical condition ▪ Clear up facts from the subject’s interpretation of the personal implications ▪ Support the subject in learning how lifestyle change may ameliorate their health and reduce cardio/metabolic/oncologic risk, using their own history ▪ Allow the subject to elicit desire and motivation to change ▪ Allow the subject to raise any questions to have tailored answers
Setting specific individual goals	▪ Allow the subject to express their reasons for change ▪ Assure the subject that they will have all the necessary support ▪ Prioritize behaviour/issues for change (patient–physician alliance) ▪ Define long-term goal/s and steps (short-term goals) to reach it/them
Education about physical activity/nutrition, tailored exercise prescription, and optimization of nutritional habits	▪ Provide knowledge and skills for exercise/nutrition ▪ Help the patient discover and define practical strategies to reduce sedentariness during their normal daily activities ▪ Help the patient to understand the role of different nutrients and their different contribution to body mass composition ▪ Ensure that the patient realizes the importance of exercise/nutrition and may be capable of appreciating any single improvement ▪ Elicit in the subject the desire to adhere to the program, taking into account medical guidelines, personal clinical needs and the subject’s preferences ▪ Explain the importance of other components of lifestyle (smoking, sleep, stress) and their relationship with exercise/nutrition habits

**Table 2:** Assessment of aerobic fitness through VO_2max_.

VO_2max_ (mL/kg/min)	Baseline (T0) Mean, sd	End Treatment (T1) Mean, sd	Pre–Post Difference: Est (95% CI)	Pre–Post Difference: t, *df*, *p*
Intention-to treat Sensitivity analysis	27.3, 6.9 26.9, 6.7	30.1, 8.0 29.4, 7.6	2.86 (2.23, 3.55) 2.45 (1.76, 3.14)	9.03, 78.3, <0.0001 * 6.95, 60.3, <0.0001 *
Per-protocol Sensitivity analysis	27.4, 7.1 27.0, 6.9	30.2, 8.0 29.5, 7.7	2.95 (2.32, 3.57) 2.45 (1.85, 3.05)	9.35, 84, <0.0001 * 8.15, 76, <0.0001 *

Estimates according to intention-to-treat principle were obtained on the complete sample (*N* = 98), using imputation methods to handle dropouts and missing values; per-protocol estimates were obtained for participants who completed the assessment of the primary outcome (*N* = 85). In both cases, sensitivity analysis was performed to assess the potential impact of the stratified sampling plan. * *p* < 0.05.

**Table 3:** Cardiopulmonary and functional outcomes at T0 and T1, with differences between time points.

Parameter	T_0_ Median, (q_25_; q_75_)	T_1_ Median, (q_25_; q_75_)	Difference Between T_1_ and T_0_ (est, 95% CI)	Difference Between T_1_ and T_0_ (Sensitivity Analysis)
Load (Watt) *	168 (127; 197)	176 (136; 224)	14.0 (7.3, 20.7)	12.0 (5.0, 21.0)
FC rest (b/min)	79 (72; 88)	79 (72; 87)	0.0 (−4.5, 4.5)	0.0 (−3.0, 6.0)
FC peak (b/min)	165 (156; 173)	167 (157; 176)	2.0 (−0.6, 4.6)	0.0 (−2.0, 4.0)
SAP rest (mmHg) *	120 (110;120)	110 (110; 116)	−10.0 (−18.3, −1.7)	−10.0 (−10.0, 0.0)
SAP peak (mmHg)	160 (150; 180)	160 (150; 170)	0.0 (−3.4, 3.4)	0.0 (0.0, 10.0)
DAP rest (mmHg)	80 (70;80)	70 (70; 80)	−5.0 (−14.7, 4.7)	−10.0 (−10.0, 0.0)
DAP peak (mmHg)	80 (80;80)	80 (70; 80)	0 (−2.5, 2.5)	0.0 (0.0, 15.0)
VE/VCO_2_ slope	26 (24; 29)	26 (24; 29)	−0.03 (−1.4, 1.1)	−0.03 (−1.91, 1.70)
VO_2_/Work(mL/min)/Watt)	9.7 (9;10)	9.8 (9.1; 10)	0.04 (−0.39, 0.43)	−0.14 (−0.73, 0.50)
AT % peak VO_2_ *	76 (68; 85)	86 (84; 88)	9.0 (4.3, 13.7)	6.0 (2.0, 16.0)
AT Load (Watt) *	123 (98; 147)	147 (112; 196)	28.0 (12.7, 43.3)	21.0 (8.0, 35.0)
AT VO_2_ (mL/min/kg) *	22 (18;25)	26 (22; 31)	4.5 (2.6, 6.4)	3.3 (1.7, 5.9)
METs peak *	8.5 (7.1; 10.0)	9.4 (8; 11)	0.7 (0.3, 1.1)	0.6 (0.3, 1.3)
6MWT (m) *	648 (604; 682)	678 (630; 720)	27.0 (13.5, 40.5)	30.0 (9.0, 57.0)
HG right arm mean (Kg)	36 (28; 43)	35 (27; 43)	0.2 (−0.8, 1.2)	0.5 (−0.9, 1.4)
HG left arm mean (Kg)	32 (26; 40)	31 (27; 40)	0.6 (−0.2, 1.5)	0.7 (−0.4, 1.9)

Data are presented as medians at enrollment (T_0_) and at the end of the exercise program (T_1_), along with the median change (T_1_ vs. T_0_). In addition, the median change obtained from the weighted analysis was presented. The number of missing observations ranged from 0 to 2 in the data at T0 and from 0 to 5 in the data at T1 and in the pre–post data. * Significant difference indicated by a 95% CI (unweighted analysis) that does not overlap with the value zero. Abbreviations: HR = heart rate; SAP = systolic arterial pressure; DAP = diastolic arterial pressure; V = volume; VE/VCO_2_ slope = relationship between ventilation and carbon dioxide production (VE vs. VCO_2_), calculated from start to the respiratory compensation point; VO_2_/Work = relationship between oxygen uptake and external work rate during CPX; AT = anaerobic threshold; O = oxygen; MET = metabolic equivalent; 6MWT = six-minute walking test; HG = handgrip. q_25_ = 25th percentile; q_75_ = 75th percentile.

**Table 4:** Body composition parameters before and after the lifestyle management program.

Parameter	T_0_ Median, (q_25_; q_75_)	T_1_ Median, (q_25_; q_75_)	Difference Between T_1_ and T_0_ (est, 95% CI)	Difference Between T1 and T0 (Sensitivity Analysis)
Height (cm)	172 (166; 177)	-	-	-
Weight (Kg)	74 (67; 90)	74 (64; 83)	−1.5 (−3.3, 0.3)	−1.0 (−2.0, 1.0)
Hydration (%)	74 (73; 74)	74 (73; 74)	0.1 (−0.06, 0.3)	0.2 (0.1, 0.6)
Waist Circumference (cm) *	92 (83; 100)	88 (79; 95)	−3.0 (−5.8, −0.2)	−2.0 (−4.0, 0.0)
BMI (kg/m^2^) *	26 (23; 28)	24 (23; 27)	−0.5 (−1.0, −0.1)	−0.4 (−0.8, 0.2)
Fat-Free Mass (Kg)	58 (48; 64)	59 (48; 65)	0.8 (−0.2, 1.8)	0.7 (−0.1, 2.2)
Fat Mass (Kg) *	19 (13; 24)	15 (11; 20)	−3.3 (−5.1, −1.5)	−2.7 (−4.7, −0.6)
Total Body Water (L)	43 (34; 48)	45 (35; 48)	0.7 (−0.01, 1.4)	0.7 (0.0, 1.3)
Body Cellular Mass (%)	30 (24; 35)	30 (25; 35)	0.2 (−0.5, 0.9)	0.3 (−0.6, 1.3)
Fat-Free Mass (%) *	75 (68; 80)	79 (74; 84)	3.7 (1.6, 5.8)	3.7 (1.2, 5.9)
Fat Mass (%) *	25 (20; 32)	21 (16; 27).	−3.7 (−5.8, −1.6)	−3.7 (−5.4, −0.4)
Total Body Water (%) *	55 (50; 59)	58 (55; 62)	2.9 (1.4, 4.4)	2.9 (0.6, 4.3)
Fat Mass Index (FMi) *	0.00069 (0.00046; 0.00085)	0.00051 (0.00038; 0.00068)	−0.00011 (−0.00018, −0.00005)	−0.000099 (−0.00017, −0.00002)
Fat-Free Mass Index (FFMi)	19 (18; 22)	19 (18; 21)	0.3 (−0.1, 0.6)	0.3 (−0.1, 0.6)

Data are presented as medians at enrollment (T_0_) and at the end of the exercise program (T_1_), along with the median change (T_1_ vs. T_0_). In addition, the median change obtained from the weighted analysis is presented. q_25_ = 25th percentile; q_75_ = 75th percentile. One missing observation at T0 and 5 or 6 missing at T1 (depending on which parameter). * Significant difference indicated by a 95% CI that does not overlap with the value zero.

## Data Availability

The datasets used and/or analyzed during the current study are available from the corresponding author on reasonable request to be assessed by the HEBE project board.

## Abbreviations

The following abbreviations are used in this manuscript:

AHA score	American Heart Association score
AT	Anaerobic threshold
BC	Body composition
BMI	Body mass index
CI	Confidence interval
CNCD	Chronic non-communicable disease
CPX	Cardiopulmonary exercise test
CRF	Cardiorespiratory Fitness
DAP	Diastolic arterial pressure
DELTA	Pre–post differences
F	Female
FM	Fat mass
FMi	Fat mass index
FFM	Fat-free mass
FFMi	Fat-free mass index
HDL	High-density lipoprotein
HG	Handgrip
HR	Heart rate
IPAQ	International physical activity questionnaire
ITT	Intention-to-treat
LDL	Low-density lipoprotein
LMPs	Lifestyle modification programs
M	Male
MAR	Missingness at random
METs	Metabolic equivalents
MET_Speak_	Metabolic equivalents at exercise peak
MICE	Multiple imputation by chain equations
MS	Metabolic syndrome
O	Oxygen
p_0_	Pre-treatment outcome proportions
p_1_	Post-treatment outcome proportions
q_25_	25th percentile
q^75^	75th percentile
RD	Relative difference
SAP	Systolic arterial pressure
T0	Baseline
T1	End of the intervention
V	Volume
VE	Ventilation
VO_2max_	Volume of maximal oxygen
W	Watt
4S-Q	Short–subjective stress-related somatic symptoms questionnaire
6MWT	Six-minute walking test
Δ	Delta

## Appendix A

### Appendix A.1. Lifestyle Assessment

- Physical activity (total activity volume) was assessed by the International Physical Activity Questionnaire (IPAQ) [36], which focuses on intensity (nominally estimated in Metabolic Equivalents (METs) according to the type of activity) and duration (in minutes) of physical activity, considering the following levels: brisk walking (≈3.3 METs), other activities of moderate intensity (≈4.0 METs) and activities of vigorous intensity (≈8.0 METs). We use the following equations to guess weekly physical activity volume: moderate-intensity [MET·minutes/week] = (3.3 × minutes of brisk walking × days of brisk walking) + (4.0 × minutes of other moderate-intensity activity × days of other moderate-intensity activities); vigorous-intensity: [MET·minutes/week] = 8.0 × minutes of vigorous-intensity activity × days of vigorous-intensity activity; and total weekly physical activity volume [MET·minutes/week] = sum of Moderate + Vigorous MET·minutes/week scores.
- We also assessed the frequency of regular strength and flexibility exercises, considering the following scale: never; sometimes; 1 section/week; 2–3 sections/week; more than 3 sections/week.
- Sedentary behaviour was assessed by asking the number of hours spent in sedentary behaviour (for instance, studying, sitting, driving, TV viewing, computer or smart devices usage) during weekly working days or weekend days.
- Nutrition was assessed using the American Heart Association Healthy Diet Score (AHA score) [37], taking into consideration fruits/vegetables, fish, sweetened beverages, whole grains, and sodium consumption.
- Sleep was assessed by inquiring about the number of hours on average slept every night and asking about the perception of sleep quality
- Stress and somatic symptom perception were assessed using a self-administered questionnaire [30,31,32,33,34,35], providing nominal self-rated scales (higher values indicate higher degrees of symptoms) focusing on: (i) the appraisal of the overall stress and fatigue perception by evaluation scales with integer scores from 0 (‘no perception’) to 10 (‘highest perception’) for each measure; (ii) the Short–Subjective Stress-related Somatic Symptoms Questionnaire (4S-Q), inquiring about four somatic symptoms accounting for the majority of somatic complaints. For scoring purposes, each response will be coded from 0 (‘no feeling’) to 10 (‘a strong feeling’); thus, the total score ranges from 0 to 40. Moreover, we also assessed the following:
- Perception of quality of sleep, quality of health, and quality of life were assessed using evaluation scales from 0 (‘worst quality) to 10 (‘best quality’) for each measure.
- Smoke behaviour: We considered non-smokers all subjects who reported having never smoked or having stopped smoking for more than one year.
- We enquired about the usage of alcohol, considering Italian habits, asking about the number of glasses of wine or beer consumed per week and the number of glasses of spirits consumed per week.
- Gum bleeding was evaluated considering the following choices: “yes”, “no”, or “sometimes”.
- Work-related discomfort was evaluated considering the following choices: “no”, “sometimes”, “yes”, or “no response”.

### Appendix A.2. Ongoing Multi-Omics Analysis and Study of the Autonomic Nervous System (ANS)

While the primary outcomes of VO_2max_ are the focus of this paper, biological samples collected during the study (blood, urine, saliva, and nasal swabs) are being analyzed as part of a comprehensive multi-omics investigation. This analysis aims to explore biomarkers of systemic inflammation and other indicators related to the study objectives, with results to be reported in future publications. Similarly, the results of the ANS will be part of future publications.

**Table A1:** Baseline description of the study subjects (*N* = 98).

Variable	
**Age (years): median (q_25_–q_75_)**	51.1 (45.0–56.2)
**Role:**	
UNIMI Faculty	38 (38.8%)
Technical and Administrative Staff	45 (45.9%)
Researcher	15 (15.3%)
**Gender: Female**	52 (53.1%)
**Education Level**	
High School Diploma	4 (4.1%)
Bachelor’s Degree	26 (26.5%)
PhD/Postgraduate Specialization	68 (59.2%)
**Frequency of medical consultations for**	
**health status assessment**	
Never	1 (1.0%)
Once per year	42 (42.9%)
Once every 2 years	3 (3.1%)
Regularly for specific medical issues	14 (14.3%)
Only when experiencing illness	38 (38.8%)
**Presence of Chronic Disease**	
No	71 (72.4%)
Yes	23 (23.5%)
No response	4 (4.1%)
**Work-related Discomfort**	
No	57 (58.2%)
Sometimes	27 (27.6%)
Yes	13 (13.3%)
No response	1 (1.0%)
**Lifestyle Goals for Improvement**	
Eating a healthier diet	22 (22.4%)
Becoming more physically active	52 (53.1%)
Managing stress	21 (21.4%)
Quitting smoking	3 (3.1%)

Age is reported as median (25th and 75th percentiles) and all other parameters as absolute number (percentage).

**Table A2:** Blood test parameters before T0 and after T1 intervention.

	T0 Median, 25th–75th Percentile Unweighted	T1 Median, 25th–75th Percentile Unweighted	Median Difference Between T1 and T0
Fasting Blood Glucose (mg/dL)	89 (85; 96)	90 (83; 95) missing: 4 (4.3%)	−2.0 (−7.3, 3.3) missing: 4 (4.3%)
Triglycerides (mg/dL)	82 (62; 118)	74 (61; 92) missing: 4 (4.3%)	−6.0 (−14.6, 2.6) missing: 4 (4.3%)
Total Cholesterol (mg/dL)	210 (179; 232)	188 (170; 218) missing: 4 (4.3%)	−8.0 (−16.2, 0.2) missing: 4 (4.3%)
HDL Cholesterol (mg/dL)	60 (50;70)	55 (50; 71) missing: 4 (4.3%)	−1.0 (−4.4, 2.4) missing: 4 (4.3%)
LDL Cholesterol (mg/dL)	124 (104; 147)	112 (96; 133) missing: 4 (4.3%)	−7.0 (−15.0, 1.0) missing: 4 (4.3%)

**Table A3:** Perception of satisfaction, difficulty and overall well-being.

**Satisfaction with the Program: median (q_25_; q_75_)**	**8 (8; 10)**
**No response**	**7 (7.6%)**
**Perceived Difficulty in Following Program:** median (q_25_; q_75_)	7 (3.3; 8)
No response	7 (7.6%)
**Overall Perception of Well-Being**	
Much Worse	0 (0.0%)
Worse	1 (1.1%)
No Change	6 (6.5%)
Improved	56 (60.9%)
Much Improved	21 (22.8%)
No response	8 (8.7%)
**Reasons for Non-Adherence * (*N* = 63)**	
Lack of Time	42 (66.7%)
Motivation Issues	7 (11.1%)
Difficulty Following the Program	4 (6.3%)
Health Issues	16 (25.4%)
Other Reasons	8 (12.7%)

* Participants were allowed to indicate one or more reasons; q_25_= 25-th, q_75_ = 75th percentiles.

**Table A4:** Differences in the other lifestyle components/parameters.

	T0 *N* (%)	T1 *N* (%)	Probability of:	Relative Difference Between T1 and T0
**Smoke** Non-smoker/ex-smoker Smokers Missing	87 (94.6%) 5 (5.4%) -	84 (91.3%) 4 (4.3%) 4 (4.3%)	Being non-ssmoker OR former smoker:	0.7% (−1.7, 3.2%)
**Gum Bleeding** No Sometime Yes Missing	79 (85.9%) 0 (0.0%) 13 (14.1%) -	79 (85.9%) 5 (5.4%) 4 (4.3%) 4 (4.3%)	No bleeding:	4.6% (−12.2%, 24.6%)
**Flexibility exercise** **(Stretching)** No sometimes 1 session/week 2–3 sessions/week More than 3 sessions/week Missing	49 (53.3%) 24 (26.1%) 8 (8.7%) 8 (8.7%) 3 (3.3%) -	26 (28.3%) 20 (21.7%) 25 (27.2%) 11 (12.0%) 6 (6.5%) 4 (4.3%)	Doing weekly stretching (any frequency)	37.2% (−3.4%, 95.0%)
**Strength Exercises** No sometimes 1 session/week 2–3 sessions/week More than 3 sessions/week Missing	21 (22.8%) 49 (53.3%) 21 (22.8%) 1 (1.1%) 0 (0.0%) -	21 (22.8%) 41 (44.6%) 26 (28.3%) 0 (0.0%) 0 (0.0%) 4 (4.3%)	Doing weekly exercise (any frequency)	3.0% (−14.7%, 24.4%)

**Table A5:** Differences in the other lifestyle components/parameters.

Parameter	T0 Median, 25th–75th Percentile Unweighted	T1 Median, 25th–75th Percentile Unweighted	Median Difference Between T1 and T0
METs TOT (MET·minutes/week)	1114 (511; 1760)	1774 (1094; 2449)	434 (44, 824)
	-	4 (4.4%)	4 (4.4%)
Sedentary behaviours (hours) *	55 (48; 65)	51 (41; 59)	−5 (−8.5, −1.5)
missing	2 (2.2%)	7 (7.8%)	9 (9.8%)
Wine/beer (glasses/week)	2 (0; 4)	1 (0; 3)	0 (−1.0, 0.0)
missing	1 (1.1%)	9 (10.0%)	10 (10.9%)
Spirits (glasses/week)	0 (0; 0)	0 (0; 0)	0 **
missing	1 (1.1%)	7 (7.8%)	8 (8.7%)
4SQ score (a.u.)	6 (2; 15)	4 (0; 12)	−1.0 (−3.7, 1.7)
missing	-	4 (5.5%)	5 (5.5%)
Stress Perception score (a.u.)	5.0 (2.8; 8.0)	4.0 (2.0; 7.0)	−1.0 (−2.6, 0.6)
missing	-	4 (4.4%)	4 (4.4%)
Fatigue Perception score (a.u.)	7.0 (3.0; 8.0)	4.0 (3.0; 8.8)	−1.0 (−2.6, 0.6)
missing	-	4 (4.4%)	4 (4.4%)
AHA healthy diet score (a.u.)	2.5 (2.0; 3.0)	3.0 (2.0; 4.0)	0.0 (−0.3, 0.3)
missing	-	4 (4.4%)	4 (4.4%)
Sleep (hours/day)	7.0 (6.0; 7.0)	7.0 (6.0; 7.0)	0 **
missing	-	4 (4.4%)	4 (4.4%)
Perception of sleep quality (a.u.)	6.0 (5.0; 8.0)	7.0 (6.0; 8.0)	0 (−0.6, 0.6)
missing	-	4 (4.4%)	4 (4.4%)
Perception of health quality (a.u.)	7.0 (6.0; 8.0)	8.0 (7.0; 8.0)	1 (−0.6, 2.6)
missing	-	4 (4.4%)	4 (4.4%)
Perception of job performance (a.u.)	7.0 (6.0; 8.0)	8.0 (7.0; 8.0)	0 (−1.2, 1.2)
missing	-	4 (4.4%)	4 (4.4%)

Abbreviations: 4SQ = Short–Subjective Stress-related Somatic Symptoms Questionnaire; AHA = American Heart Association; a.u. = arbitrary unis; * significant difference indicated by a 95% CI that does not overlap with the value zero; ** confidence intervals not reported, due to unreliable estimate of the standard error of the median with quantile regression method and/or weighted methods (due to the fact no change was observed in sleep hours for 48 over 85 participants, and no change in spirit consumption for 67/85 participants).

**Table A6:** Distribution of categorical variables in completer and non-completer groups.

Parameter	Completers (*n* = 92)	Non-Completers (*n* = 6)	Cramer V
**Gender**			0.404
Female	51 (55.4%)	1 (16.7%)	
Male	41 (44.6%)	5 (83.3%)	
**Physical activity**			0.082
**<**600 MET minutes/week	54 (58.7%)	4 (66.7%)	
**≥**600 MET minutes/week	38 (41.3%)	2 (33.3%)	
**AHA score**			0.188
0–1	16 (17.4%)	2 (33.3%)	
2–3	60 (65.2%)	3 (50.0%)	
4–5	16 (17.4%)	1 (16.7%)	
**Metabolic syndrome**			0.093
No	69 (75.8%)	5 (83.3%)	
Yes	22 (24.2%)	1 (16.7%)	

Abbreviations: MET = metabolic equivalent; AHA = American Heart Association.

## References

[1] Bull F.C., Al-Ansari S.S., Biddle S., Borodulin K., Buman M.P., Cardon G., Carty C., Chaput J.P., Chastin S., Chou R. (2020). World Health Organization 2020 Guidelines on Physical Activity and Sedentary Behaviour. Br. J. Sports Med..

[2] Sallis R.E. (2009). Exercise Is Medicine and Physicians Need to Prescribe It!. Br. J. Sports Med..

[3] Pelliccia A., Sharma S., Gati S., Bäck M., Börjesson M., Caselli S., Collet J.P., Corrado D., Drezner J.A., Halle M. (2021). 2020 ESC Guidelines on Sports Cardiology and Exercise in Patients with Cardiovascular Disease. Eur. Heart J..

[4] Valenzuela P.L., Ruilope L.M., Santos-Lozano A., Wilhelm M., Kränkel N., Fiuza-Luces C., Lucia A. (2023). Exercise Benefits in Cardiovascular Diseases: From Mechanisms to Clinical Implementation. Eur. Heart J..

[5] Oppert J.M., Bellicha A., Ciangura C. (2021). Physical Activity in Management of Persons with Obesity. Eur. J. Intern. Med..

[6] Argentieri M.A., Amin N., Nevado-Holgado A.J., Sproviero W., Collister J.A., Keestra S.M., Kuilman M.M., Ginos B.N.R., Ghanbari M., Doherty A. (2025). Integrating the Environmental and Genetic Architectures of Aging and Mortality. Nat. Med..

[7] Silverman M.N., Deuster P.A. (2014). Biological Mechanisms Underlying the Role of Physical Fitness in Health and Resilience. Interface Focus.

[8] Nieman D.C., Wentz L.M. (2019). The Compelling Link between Physical Activity and the Body’s Defense System. J. Sport Health Sci..

[9] Fiuza-Luces C., Valenzuela P.L., Gálvez B.G., Ramírez M., López-Soto A., Simpson R.J., Lucia A. (2024). The Effect of Physical Exercise on Anticancer Immunity. Nat. Rev. Immunol..

[10] Bishop V.S. (2004). Exercise and the Autonomic Nervous System. Primer on the Autonomic Nervous System.

[11] Kokkinos P., Faselis C., Samuel I.B.H., Lavie C.J., Zhang J., Vargas J.D., Pittaras A., Doumas M., Karasik P., Moore H. (2023). Changes in Cardiorespiratory Fitness and Survival in Patients With or Without Cardiovascular Disease. J. Am. Coll. Cardiol..

[12] Kodama S., Saito K., Tanaka S., Maki M., Yachi Y., Asumi M., Sugawara A., Totsuka K., Shimano H., Ohashi Y. (2009). Cardiorespiratory Fitness as a Quantitative Predictor of All-Cause Mortality and Cardiovascular Events in Healthy Men and Women: A Meta-Analysis. JAMA.

[13] Cumming G.R. (1968). Physical Fitness and Cardiovascular Health. Circulation.

[14] Lang J.J., Prince S.A., Merucci K., Cadenas-Sanchez C., Chaput J.P., Fraser B.J., Manyanga T., McGrath R., Ortega F.B., Singh B. (2024). Cardiorespiratory Fitness Is a Strong and Consistent Predictor of Morbidity and Mortality among Adults: An Overview of Meta-Analyses Representing over 20.9 Million Observations from 199 Unique Cohort Studies. Br. J. Sports Med..

[15] Nilsson M.I., Bourgeois J.M., Nederveen J.P., Leite M.R., Hettinga B.P., Bujak A.L., May L., Lin E., Crozier M., Rusiecki D.R. (2019). Lifelong Aerobic Exercise Protects against Inflammaging and Cancer. PLoS ONE.

[16] Woods J.A., Wilund K.R., Martin S.A., Kistler B.M. (2011). Exercise, Inflammation and Aging. Aging Dis..

[17] Rippe J.M., Frates B. (2025). Academic Lifestyle Medicine and Physician Education. Am. J. Lifestyle Med..

[18] Stead M., Craigie A.M., Macleod M., McKell J., Caswell S., Steele R.J.C., Anderson A.S. (2015). Why Are Some People More Successful at Lifestyle Change than Others? Factors Associated with Successful Weight Loss in the BeWEL Randomised Controlled Trial of Adults at Risk of Colorectal Cancer. Int. J. Behav. Nutr. Phys. Act..

[19] Murray J., Craigs C.L., Hill K.M., Honey S., House A. (2012). A Systematic Review of Patient Reported Factors Associated with Uptake and Completion of Cardiovascular Lifestyle Behaviour Change. BMC Cardiovasc. Disord..

[20] Lucini D., Pagani M. (2021). Exercise Prescription to Foster Health and Well-Being: A Behavioral Approach to Transform Barriers into Opportunities. Int. J. Environ. Res. Public Health.

[21] Friedberg M.W., Schneider E.C., Rosenthal M.B., Volpp K.G., Werner R.M. (2014). Association between Participation in a Multipayer Medical Home Intervention and Changes in Quality, Utilization, and Costs of Care. JAMA.

[22] Song Z., Baicker K. (2019). Effect of a Workplace Wellness Program on Employee Health and Economic Outcomes: A Randomized Clinical Trial. JAMA.

[23] Ross R., Blair S.N., Arena R., Church T.S., Després J.P., Franklin B.A., Haskell W.L., Kaminsky L.A., Levine B.D., Lavie C.J. (2016). Importance of Assessing Cardiorespiratory Fitness in Clinical Practice: A Case for Fitness as a Clinical Vital Sign: A Scientific Statement from the American Heart Association. Circulation.

[24] Wasserman K., Van Kessel A.L., Burton G.G. (1967). Interaction of Physiological Mechanisms during Exercise. J. Appl. Physiol..

[25] Kokkinos P., Faselis C., Samuel I.B.H., Pittaras A., Doumas M., Murphy R., Heimall M.S., Sui X., Zhang J., Myers J. (2022). Cardiorespiratory Fitness and Mortality Risk Across the Spectra of Age, Race, and Sex. J. Am. Coll. Cardiol..

[26] Fletcher G.F., Ades P.A., Kligfield P., Arena R., Balady G.J., Bittner V.A., Coke L.A., Fleg J.L., Forman D.E., Gerber T.C. (2013). Exercise Standards for Testing and Training: A Scientific Statement from the American Heart Association. Circulation.

[27] Bianchi F., Biganzoli E.M., Bollati V., Clerici M., Lucini D., Mandò C., Rota F. (2024). HEBE Project: Healthy Aging versus Inflamm-Aging: The Role of Physical Exercise in Modulating the Biomarkers of Age-Associated and Environmentally Determined Chronic Diseases, Study Protocol. PLoS ONE.

[28] Grundy S.M., Hansen B., Smith S.C., Cleeman J.I., Kahn R.A. (2004). Clinical Management of Metabolic Syndrome: Report of the American Heart Association/National Heart, Lung, and Blood Institute/American Diabetes Association Conference on Scientific Issues Related to Management. Arterioscler. Thromb. Vasc. Biol..

[29] Benton M.J., Swan P.D., Schlairet M.C., Sanderson S. (2011). Comparison of Body Composition Measurement With Whole Body Multifrequency Bioelectrical Impedance and Air Displacement Plethysmography in Healthy Middle-Aged Women. Health Care Women Int..

[30] Lucini D., Riva S., Pizzinelli P., Pagani M. (2007). Stress Management at the Worksite: Reversal of Symptoms Profile and Cardiovascular Dysregulation. Hypertension.

[31] Lucini D., Luconi E., Giovanelli L., Marano G., Bernardelli G., Guidetti R., Morello E., Cribellati S., Brambilla M.M., Biganzoli E.M. (2024). Assessing Lifestyle in a Large Cohort of Undergraduate Students: Significance of Stress, Exercise and Nutrition. Nutrients.

[32] Solaro N., Oggionni G., Bernardelli G., Malacarne M., Pagani E., Ferrari M., Parati G., Lucini D. Lifestyle and Perceived Well-Being in Children and Teens: Importance of Exercise and Sedentary Behavior. Nutrients **2370**.

[33] Lucini D., Malacarne M., Gatzemeier W., Pagani E., Bernardelli G., Parati G., Pagani M. (2022). Evidence of Better Autonomic, Metabolic and Psychological Profile in Breast Cancer Survivors Meeting Current Physical Activity Recommendations: An Observational Study. J. Pers. Med..

[34] Lucini D., Malacarne M., Gatzemeier W., Pagani M. (2020). A Simple Home-Based Lifestyle Intervention Program to Improve Cardiac Autonomic Regulation in Patients with Increased Cardiometabolic Risk. Sustainability.

[35] Lucini D., Solaro N., Lesma A., Gillet V.B., Pagani M. (2011). Health Promotion in the Workplace: Assessing Stress and Lifestyle with an Intranet Tool. J. Med. Internet Res..

[36] Craig C.L., Marshall A.L., Sjöström M., Bauman A.E., Booth M.L., Ainsworth B.E., Pratt M., Ekelund U., Yngve A., Sallis J.F. (2003). International Physical Activity Questionnaire: 12-Country Reliability and Validity. Med. Sci. Sports Exerc..

[37] Lloyd-Jones D.M., Hong Y., Labarthe D., Mozaffarian D., Appel L.J., Van Horn L., Greenlund K., Daniels S., Nichol G., Tomaselli G.F. (2010). Defining and Setting National Goals for Cardiovascular Health Promotion and Disease Reduction: The American Heart Association’s Strategic Impact Goal through 2020 and Beyond. Circulation.

[38] Rost R., Hollmann W. (1982). Belastungsuntersuchungen in Der Praxis: Grundlagen, Technik Und Interpretation Ergometrischer Untersuchungsverfahren.

[39] Agarwala P., Salzman S.H. (2020). Six-Minute Walk Test: Clinical Role, Technique, Coding, and Reimbursement. Chest.

[40] Tomkinson G.R., Lang J.J., Rubín L., McGrath R., Gower B., Boyle T., Klug M.G., Mayhew A.J., Blake H.T., Ortega F.B. (2024). International Norms for Adult Handgrip Strength: A Systematic Review of Data on 2.4 Million Adults Aged 20 to 100+ Years from 69 Countries and Regions. J. Sport Health Sci..

[41] Maslov P.Z., Schulman A., Lavie C.J., Narula J. (2018). Personalized Exercise Dose Prescription. Eur. Heart J..

[42] Mozaffarian D. (2016). Dietary and Policy Priorities for Cardiovascular Disease, Diabetes, and Obesity: A Comprehensive Review. Circulation.

[43] Kurnik Mesarič K., Pajek J., Logar Zakrajšek B., Bogataj Š., Kodrič J. (2023). Cognitive Behavioral Therapy for Lifestyle Changes in Patients with Obesity and Type 2 Diabetes: A Systematic Review and Meta-Analysis. Sci. Rep..

[44] Spring B., Ockene J.K., Gidding S.S., Mozaffarian D., Moore S., Rosal M.C., Brown M.D., Vafiadis D.K., Cohen D.L., Burke L.E. (2013). Better Population Health through Behavior Change in Adults: A Call to Action. Circulation.

[45] Friedel A.L., Siegel S., Kirstein C.F., Gerigk M., Bingel U., Diehl A., Steidle O., Haupeltshofer S., Andermahr B., Chmielewski W. (2023). Measuring Patient Experience and Patient Satisfaction-How Are We Doing It and Why Does It Matter? A Comparison of European and U.S. American Approaches. Healthcare.

[46] White I.R., Horton N.J., Carpenter J., Pocock S.J. (2011). Strategy for Intention to Treat Analysis in Randomised Trials with Missing Outcome Data. BMJ.

[47] Rubin D.B. (1987). Multiple Imputation for Nonresponse in Surveys.

[48] White I.R., Royston P., Wood A.M. (2011). Multiple Imputation Using Chained Equations: Issues and Guidance for Practice. Stat. Med..

[49] Fitzmaurice M., Laird N., Ware J. (2011). Applied Longitudinal Analysis.

[50] Heeringa S.G., West B.T., Berglund P.A. (2010). Applied Survey Data Analysis.

[51] Des Jarlais D.C., Lyles C., Crepaz N. (2004). Improving the Reporting Quality of Nonrandomized Evaluations of Behavioral and Public Health Interventions: The TREND Statement. Am. J. Public Health.

[52] Koenker R. (2005). Quantile Regression. Quantile Regression.

[53] R Foundation for Statistical Computing, Vienna R: A Language and Environment for Statistical Computing. https://www.r-project.org/.

[54] Huntington-Klein N. Variable Table for Variable Documentation • Vtable. https://nickch-k.github.io/vtable/.

[55] van Buuren S., Groothuis-Oudshoorn K. (2011). Mice: Multivariate Imputation by Chained Equations in R. J. Stat. Softw..

[56] Lumley T. (2004). Analysis of Complex Survey Samples. J. Stat. Softw..

[57] Deslippe A.L., Soanes A., Bouchaud C.C., Beckenstein H., Slim M., Plourde H., Cohen T.R. (2023). Barriers and Facilitators to Diet, Physical Activity and Lifestyle Behavior Intervention Adherence: A Qualitative Systematic Review of the Literature. Int. J. Behav. Nutr. Phys. Act..

[58] Kulmala J., Rosenberg A., Ngandu T., Hemiö K., Tenkula T., Hyytiä A., Vienola M., Huhtamäki-Kuoppala M., Saarinen A., Korkki S. (2021). Facilitators and Barriers to Implementing Lifestyle Intervention Programme to Prevent Cognitive Decline. Eur. J. Public Health.

[59] Narducci D.M. (2023). Black Box Warning: When Exercise Is Not Medicine. Br. J. Sports Med..

[60] Schumacher B.T., Di C., Bellettiere J., Lamonte M.J., Simonsick E.M., Parada H., Hooker S.P., Lacroix A.Z. (2023). Validation, Recalibration, and Predictive Accuracy of Published VO_2max_ Prediction Equations for Adults Ages 50–96 Yr. Med. Sci. Sports Exerc..

[61] Campodonico J., Willixhofer R., Salvioni E., Rangel L.G.G., Benitez-Perez R.E., Mapelli M., Agostoni P. (2025). Ventilation Efficiency during Exercise: The Delicate Balance behind Carbon Dioxide Removal. Eur. J. Prev. Cardiol..

[62] Kowalski T., Kasiak P., Chomiuk T., Mamcarz A., Śliż D. (2025). Optimizing the Interpretation of Cardiopulmonary Exercise Testing in Endurance Athletes: Precision Approach for Health and Performance. Transl. Sports Med..

[63] Peterman J.E., Arena R., Myers J., Marzolini S., Ades P.A., Savage P.D., Lavie C.J., Kaminsky L.A. (2021). Reference Standards for Cardiorespiratory Fitness by Cardiovascular Disease Category and Testing Modality: Data from Friend. J. Am. Heart Assoc..

[64] Zheng P., Yan W., Ding Y., Zhang Y., Chen Z., Qian J., Ge J. (2026). Cardiovascular Ageing: Hallmarks, Signaling Pathways, Diseases and Therapeutic Targets. Signal Transduct. Target. Ther..

[65] Ozaki H., Loenneke J.P., Thiebaud R.S., Abe T. (2013). Resistance Training Induced Increase in VO2max in Young and Older Subjects. Eur. Rev. Aging Phys. Act..

[66] Bacon A.P., Carter R.E., Ogle E.A., Joyner M.J. (2013). VO2max Trainability and High Intensity Interval Training in Humans: A Meta-Analysis. PLoS ONE.

[67] Crowley E., Powell C., Carson B.P., Davies R.W. (2022). The Effect of Exercise Training Intensity on VO2max in Healthy Adults: An Overview of Systematic Reviews and Meta-Analyses. Transl. Sports Med..

[68] McCarthy D., Berg A. (2021). Weight Loss Strategies and the Risk of Skeletal Muscle Mass Loss. Nutrients.

[69] Martin S.S., Aday A.W., Allen N.B., Almarzooq Z.I., Anderson C.A.M., Arora P., Avery C.L., Baker-Smith C.M., Bansal N., Beaton A.Z. (2025). 2025 Heart Disease and Stroke Statistics: A Report of US and Global Data From the American Heart Association. Circulation.

[70] Varela A.J., Gallamore M.J., Hansen N.R., Martin D.C. (2024). Patient Empowerment: A Critical Evaluation and Prescription for a Foundational Definition. Front. Psychol..

[71] Laddu D., Neeland I.J., Carnethon M., Stanford F.C., Mongraw-Chaffin M., Gibbs B.B., Ndumele C.E., Longenecker C.T., Chung M.L., Rao G. (2024). Implementation of Obesity Science Into Clinical Practice: A Scientific Statement From the American Heart Association. Circulation.

[72] Kris-Etherton P.M., Petersen K.S., Després J.P., Anderson C.A.M., Deedwania P., Furie K.L., Lear S., Lichtenstein A.H., Lobelo F., Morris P.B. (2021). Strategies for Promotion of a Healthy Lifestyle in Clinical Settings: Pillars of Ideal Cardiovascular Health: A Science Advisory from the American Heart Association. Circulation.

[73] Buja A., Rabensteiner A., Sperotto M., Grotto G., Bertoncello C., Cocchio S., Baldovin T., Contu P., Lorini C., Baldo V. (2020). Health Literacy and Physical Activity: A Systematic Review. J. Phys. Act. Health.

[74] Lorig K.R., Holman H.R. (2003). Self-Management Education: History, Definition, Outcomes, and Mechanisms. Ann. Behav. Med..

[75] Unlike Less Educated People, College Grads More Active on Weekends than Weekdays|ScienceDaily. https://www.sciencedaily.com/releases/2014/08/140819082914.htm#google_vignette.

[76] Stress Management|American Heart Association. https://www.heart.org/en/healthy-living/healthy-lifestyle/stress-management.

[77] Stress. https://www.who.int/news-room/questions-and-answers/item/stress.

[78] Park S., Jang M.K. (2019). Associations Between Workplace Exercise Interventions and Job Stress Reduction: A Systematic Review. Workplace Health Saf..

[79] Schuch F.B., Stubbs B., Meyer J., Heissel A., Zech P., Vancampfort D., Rosenbaum S., Deenik J., Firth J., Ward P.B. (2019). Physical Activity Protects from Incident Anxiety: A Meta-Analysis of Prospective Cohort Studies. Depress. Anxiety.

[80] Zeng W., Stason W.B., Fournier S., Razavi M., Ritter G., Strickler G.K., Bhalotra S.M., Shepard D.S. (2013). Benefits and Costs of Intensive Lifestyle Modification Programs for Symptomatic Coronary Disease in Medicare Beneficiaries. Am. Heart J..

[81] Alencar M., Sauls R., Whetten J. (2026). Cost-Effectiveness of a Lifestyle and Behavioral Care Model Targeting Cardiometabolic Disease Progression. Int. J. Environ. Res. Public Health.

[82] Hajat C., Kotzen D., Stein E., Yach D. (2019). Physical Activity Is Associated with Improvements in Other Lifestyle Behaviours. BMJ Open Sport Exerc. Med..

